# Zika virus and temperature modulate *Elizabethkingia anophelis* in *Aedes albopictus*

**DOI:** 10.1186/s13071-021-05069-7

**Published:** 2021-11-12

**Authors:** Maria G. Onyango, Rachel Lange, Sean Bialosuknia, Anne Payne, Nicholas Mathias, Lili Kuo, Aurelien Vigneron, Dilip Nag, Laura D. Kramer, Alexander T. Ciota

**Affiliations:** 1 Department of Biological Sciences, College of Arts and Sciences, Texas Tech University, 2901 Main St, Lubbock, TX 79409 USA; 2 Arbovirus Laboratory, Wadsworth Center, New York State Department of Health, 5668 State Farm Road, Slingerlands, NY 12159 USA; 3 Department of Biomedical Sciences, School of Public Health, State University of New York at Albany, 1400 Washington Avenue, Rensselaer, NY 12144 USA; 4 Institute of Organismic and Molecular Evolution, Johannes Gutenberg University Mainz, 55128 Mainz, Germany

**Keywords:** Zika virus, *Aedes albopictus*, Microbiome, *Elizabethkingia*

## Abstract

**Background:**

Vector-borne pathogens must survive and replicate in the hostile environment of an insect’s midgut before successful dissemination. Midgut microbiota interfere with pathogen infection by activating the basal immunity of the mosquito and by synthesizing pathogen-inhibitory metabolites.

**Methods:**

The goal of this study was to assess the influence of Zika virus (ZIKV) infection and increased temperature on *Aedes albopictus* midgut microbiota. *Aedes albopictus* were reared at diurnal temperatures of day 28 °C/night 24 °C (L) or day 30 °C/night 26 °C (M). The mosquitoes were given infectious blood meals with 2.0 × 10^8^ PFU/ml ZIKV, and 16S *rRNA* sequencing was performed on midguts at 7 days post-infectious blood meal exposure.

**Results:**

Our findings demonstrate that *Elizabethkingia anophelis albopictus* was associated with *Ae. albopictus* midguts exposed to ZIKV infectious blood meal. We observed a negative correlation between ZIKV and *E. anophelis albopictus* in the midguts of *Ae. albopictus*. Supplemental feeding of *Ae. albopictus* with *E. anophelis aegypti* and ZIKV resulted in reduced ZIKV infection rates. Reduced viral loads were detected in Vero cells that were sequentially infected with *E. anophelis aegypti* and ZIKV, dengue virus (DENV), or chikungunya virus (CHIKV).

**Conclusions:**

Our findings demonstrate the influence of ZIKV infection and temperature on the *Ae. albopictus* microbiome along with a negative correlation between ZIKV and *E. anophelis albopictus*. Our results have important implications for controlling vector-borne pathogens.

**Graphical Abstract:**

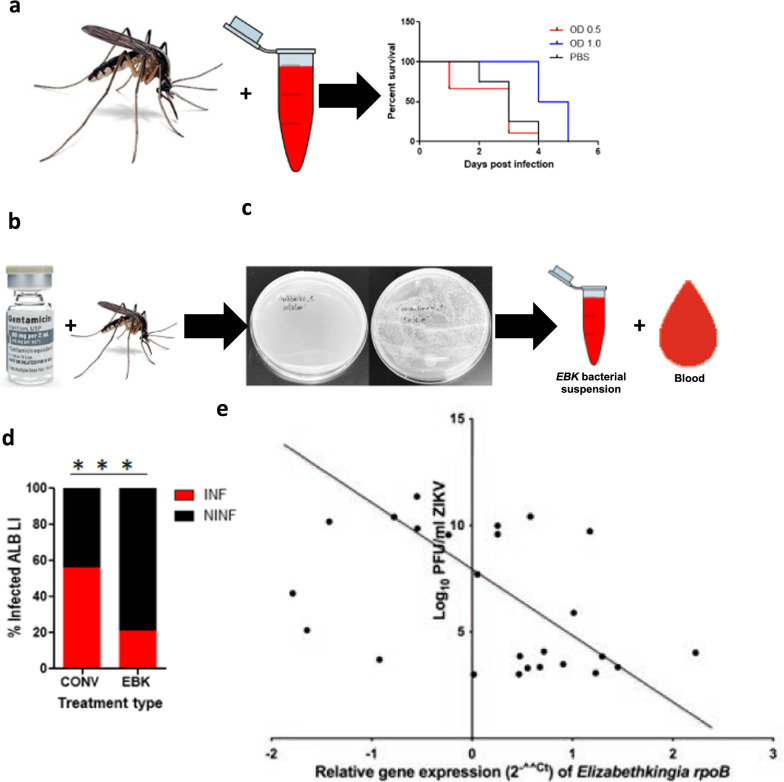

**Supplementary Information:**

The online version contains supplementary material available at 10.1186/s13071-021-05069-7.

## Background

Zika virus (ZIKV), a *Flavivirus* of the family *Flaviviridae* with an ~ 11-kb positive-sense, single-stranded RNA genome [1], is classified as a member of the Spondweni group [2]. There are two main ZIKV lineages: one lineage with two genotypes from Africa and another from Asia [3, 4].

ZIKV is mainly transmitted by the bite of infected *Aedes* mosquitoes, although sexual, transplacental, and blood transfusion transmissions have also been documented [5]. There is no Food and Drug Administration (FDA)-approved medication or vaccine to treat or prevent ZIKV infection, despite the World Health Organization (WHO) declaring ZIKV a public health emergency of international concern [6]. Medications for symptomatic relief are the only source of relief for infected individuals [7]. Control measures based on preventing sexual transmission and controlling the mosquito vector are the main prevention strategies against ZIKV [5]. An upsurge in insecticide resistance, as well as climate change, has expanded the geographical range of vector territories [8], highlighting the urgent need for alternative mosquito control approaches.

The interaction of gut microbial taxa and arboviruses of medical importance has been studied extensively [9–17] due to the potential for microbes to act as candidates for the development of novel therapeutic and transmission-blocking agents.

Flavobacteria have been shown to dominate the *Anopheles* mosquito midgut [18]. *Elizabethkingia* (*EBK*)—a gram-negative, rod-like, aerobic, non-fermenting, non-motile, and non-spore-forming flavobacterium—is widely distributed in *Aedes* [18–20] and *Anopheles* [21–23] mosquitoes. *EBK* has been isolated from both field-caught and laboratory-reared mosquitoes in Africa, Europe, and North America [20, 23, 24] and can be transmitted among mosquitoes through vertical, transstadial, or horizontal transmission [18, 22]. In addition, *Elizabethkingia meningoseptica* metabolites obtained from *Anopheles stephensi* midguts possess antiparasitic effects, as well as gametocyte toxicity [25]. Similarly, *Elizabethkingia anophelis* has a significant effect on *Plasmodium* parasite development, reducing the oocyst load when introduced at a low bacterial dose [21].

A previous metagenomics study aimed at understanding the effect of ZIKV on the dynamic bacterial community harbored by *Aedes aegypti* [26] demonstrated that midgut microbes belonging to the family *Flavobacteriaceae* were the most abundant bacterial taxa among ZIKV-infected mosquitoes. This was particularly the case among the ZIKV-exposed, blood-fed individuals. *Flavobacteriaceae* abundance was high during the first days of infection but decreased at 7 days post-infection (dpi) among ZIKV-exposed gravid individuals [26]. The aims of this study were to identify the bacterial species within the *Aedes albopictus* midgut that significantly correlate with ZIKV infectivity and replication, assess whether these microbes possessed antiviral properties, and characterize the influence of temperature on these interactions. Once identified, the symbionts associated with altered vector competence can potentially inform novel vector-borne pathogen control measures.

## Methods

### Virus

The ZIKV strain used in this study was HND 2016–19563 (GenBank accession no. KX906952), which was isolated in 2016 in New York state from an infected patient who had traveled to Honduras [27]. To create stock virus for this study, the virus was isolated and passaged three times on Vero cell culture and once on C6/36 cell culture. Stocks were frozen at −70 °C prior to use. Chikungunya virus (CHIKV) IDR 140025961 was isolated from a patient in the Dominican Republic in 2014, while the dengue virus serotype 2 (DENV-2) NI2B strain 306 was isolated from a patient in Nicaragua in 2007. All virus samples were treated similarly.

### Mosquito sampling and screening for ZIKV infection

The Intergovernmental Panel on Climate Change (IPCC) predicts a 2–4 °C mean global temperature rise over the next century due to global warming [28]. We modeled a 2 °C increase (M temperature regimen) over our baseline temperatures (L temperature regimen), representing the mean temperatures during peak transmission in Suffolk County (NY), where the *Ae. albopictus* were collected in 2015 (kindly provided by I. Rochlin). *Ae. albopictus* were colonized at the New York State Department of Health (NYSDOH) Arbovirus Laboratory.

The 15th generation following laboratory colonization of *Ae. albopictus* were vacuum-hatched and maintained at 28 °C under standard rearing conditions.

To initiate the experiment aimed at exposing them to ZIKV, the mosquitoes were hatched and reared under two thermal conditions: 14 h light at 30 °C/10 h dark at 26 °C (M temperature regimen), or 14 h light at 28 °C/10 h dark at 24 °C (L temperature regimen).

At 8 days post-eclosion, female adults were orally exposed to blood meals consisting of either 1:1 dilution of defibrinated sheep blood plus 2.5% sucrose, sodium bicarbonate (to adjust pH to 8.0) and the virus, or a non-infectious blood meal (NIBM) containing a final concentration of 2.5% sucrose solution. Infectious blood meals contained 2.00E+08 PFU/ml ZIKV HND [27]. The female mosquitoes were exposed to blood meals in a 37 °C preheated Hemotek membrane feeding system (Discovery Workshops, Accrington, UK) with a porcine sausage casing membrane. After 1 h, the mosquitoes were anesthetized with CO_2_ and immobilized on a pre-chilled tray connected to CO_2._ Engorged females were separated and placed in three separate 0.6 L cardboard cartons (30 individuals/carton). Blood-fed females were maintained on 10% sucrose solution provided ad libitum.

At 7 dpi, the female mosquitoes were immobilized using triethylamine (Sigma-Aldrich, St. Louis, MO, USA). To examine for ZIKV dissemination in the mosquito, the legs were removed before dissecting the gut. To assess transmission, saliva was collected under sterile conditions by inserting the proboscis of each female mosquito into a capillary tube containing ~ 20 µl fetal bovine serum (FBS) plus 50% sucrose 1:1 for 30 min. The mixture was then ejected into 125 µl of mosquito diluent (MD) (20% heat-inactivated FBS in Dulbecco phosphate-buffered saline plus 50 µg/ml penicillin/streptomycin, 50 µg/ml gentamicin, and 2 µg/ml Fungizone [Sigma-Aldrich, St. Louis, MO, USA]). The midgut was dissected from surface-sterilized individual mosquitoes and rinsed twice in sterile phosphate-buffered saline (PBS) before transfer to sterile microcentrifuge tubes and storage at −80 °C until testing. Mosquito carcass and legs were then placed in individual tubes containing 500 µl MD and a bead. All samples were held at −80 °C until assaying. An absolute quantity of ZIKV in the legs, carcass, and saliva was obtained by isolating RNA using TRIzol (Thermo Fisher Scientific, Waltham, MA, USA). A ZIKV-specific quantitative polymerase chain reaction (qPCR) assay targeting the NS1 region was used for detection (Table 1). Serially diluted ZIKV of known quantities was used to generate the standard curve and extrapolate viral load (Additional file 1: Figure S1). Midguts obtained from *Ae. albopictus* adults maintained on 10% sucrose solution were used as a control.

**Table 1:** Primer sequences and reaction conditions

Primer name	Primer sequence	Annealing temperature (°C)
Ribosomal protein S7_F	GAG ATC GAG TTC AAC AGC AAG A	55
Ribosomal protein S7_R	GAG AAC TTC TTC TCC AGC TCA C	
*Elizabethkingia*_rpoB_F	CTC CGG AAG GAC CAA ACA TTG	55
*Elizabethkingia*_rpoB_R	CAA CCG TCC AGT CAG ATC C	
*Elizabethkingia*_qrpoB_F	GCT AGG ACA CAT GGT TGA TGA	55
*Elizabethkingia*_qrpoB_R	ACC ACC GAA CTG AGC TTT AC	
16S Microbiome_F	TCG TCG GCA GCG TCA GAT GTG TAT AAG AGA CAG CCT ACG GGN GGC WGC AG	55
16S Microbiome_R	GTC TCG TGG GCT CGG AGA TCT GTA TAA GAG ACA GGA CTA CHV GGG TAT CTA ATC C	
*ChikF1*	AAGCTCCGCGTCCTTTACCAAG	60
*ChikR1*	CCAAATTGTTCCTGGTCTTCCT	
*Chik Probe* [60, 61]	CCAATGTCTTCAGCCTGGACACCTTT	
*DenF1*	AAGGACTAGAGGTTAKAGGAGACCC	60
*DenR1*	GGCGYTCTGTGCCTGGAWTGATG	
*Den Probe 1–3* [62]	AACAGCATATTGACGCTGGGAGAGACC	
*ZIKV 1086*	CCGCTGCCCAACACAAG	60
*ZIKV 1162c*	CCACTAACGTTCTTTTGCAGACAT	
*ZIKV 1107-FAM* [4]	AGCCTACCTTGACAAGCAGTCAGACACTCAA	

### Sequencing and analysis of microbiome of *Aedes* mosquito midguts

A total of 29 samples were included in this study: 10 midguts exposed to infected blood meal (IBM), 10 midguts exposed to NIBM, and nine midguts exposed to 10% sucrose only (non-blood-fed [NBF]), along with one negative control (NC), one non-template control (NTC), and one positive spike (PC). *Aedes albopictus* midgut, legs, carcass, and saliva samples were screened for virus at 7 dpi. The infection and dissemination status of each individual was determined prior to downstream processing. The complementary DNA (cDNA) from individual midguts was prepared from the RNA samples using the iScript cDNA kit (Bio-Rad, Hercules, CA, USA). Thereafter, 16S *rRNA* was amplified using the 16S primer set and conditions from Table 1.

The barcoded high-throughput amplicon sequencing of the bacterial 16S V3-V4 hypervariable *rRNA* was completed at Wadsworth Center’s Advanced Genomic Technologies Core (WCAGT). PCR reactions were carried out in a total volume of 50 µl: 5 µM of 16S *rRNA* V3-V4 hypervariable region primers consisting of an Illumina barcode (Table 1), 2 µl cDNA, 8 µl deionized filter sterilized water, and 36 µl AccuStart II PCR SuperMix (Quanta Biosciences, Beverly, MA, USA). To ensure identification of any potential contamination at either the PCR stage or the sequencing step, two negative controls (water and non-template control) were included at the PCR step and sequenced. A positive spike was included during the sequencing run. A fragment size of ~ 460 base pairs (bp) of each sample, as well as the negative controls, was submitted to the WCAGT for sequencing. Automated cluster generation and paired-end sequencing (250-bp reads) were performed using the Illumina MiSeq 500-cycle reagent kit.

Data analysis was carried out on QIITA (https://qiita.ucsd.edu/) and MicrobiomeAnalyst [29]. In summary, to convert the de-multiplexed FASTQ files to the format used by QIITA, the command split libraries FASTQ was used. The clustering of the sequences into operational taxonomic units (OTUs) was done via closed-reference OTU picking based on the Greengenes 16S reference database, which matches the sequences based on a 97% sequence identity threshold before assigning taxonomies. A BIOM-formatted OTU table was subsequently generated, and in order to reduce the potential for alpha and beta diversity biases, the data were rarefied to a depth of 30,000 reads per sample. Finally, to visualize the taxonomic profiles of each sample, taxa bar plots were generated using the rarefied data artifact. Following the generation of the relative quantity of each taxon, we generated taxa which were deposited in the NCBI GenBank Short Read Archives with accession numbers PRJNA608507, PRJNA608505, PRJNA608503, PRJNA608501, PRJNA608500, PRJNA608499, PRJNA608472, PRJNA608496, PRJNA608495, PRJNA608493, PRJNA608492, PRJNA608491, PRJNA608489, PRJNA608483, PRJNA608471, PRJNA608482, PRJNA608470, PRJNA608456, PRJNA608450, PRJNA608481, PRJNA608449, PRJNA608448, PRJNA608480, PRJNA608479, PRJNA608477, PRJNA608437, PRJNA608434, PRJNA608427, and PRJNA608428.

The alpha diversity profiling (number of species of taxa in a single sample) was calculated using the Chao1 diversity measure, and significance testing was done using analysis of variance (ANOVA) in MicrobiomeAnalyst. The Chao1 index estimates the richness of taxa in a sample by extrapolating the number of rare organisms that may have been omitted due to under-sampling. The index assumes that the number of observations for a taxon has a Poisson distribution and thus corrects for variance [30]. Beta diversity, a comparison of microbial community composition between two samples or two conditions, was analyzed by utilizing the Bray–Curtis dissimilarity statistic [31]. The distance matrix generated after comparing each sample to the other was visualized for dissimilarity between samples using the principal coordinates analysis method.

### Phylogenetic analysis of the *Elizabethkingia* (*EBK*) isolated from *Ae. albopictus* midguts

In order to validate the identity of *EBK* taxa identified in the *Ae. albopictus* midguts, the *rpoB* gene (accession number MT096047) was amplified from the midgut cDNA using the *rpoB* primer pairs and PCR conditions as described in Table 1. The 558-bp PCR amplicon was excised from an agarose gel and cleaned using the QIAquick gel extraction kit (Qiagen, UK). The amplicons were Sanger-sequenced at the WCAGT. To construct the *EBK rpoB* phylogenetic tree, Molecular Evolutionary Genetic Analysis (MEGA) software [32] was utilized.

A total of nine gene sequences published with GenBank accession numbers CP023010.2, KY587659.1, CP016377.1, KY587657.1, CP035811.1, CP035809.1, CP011059.1, CP039929.1, and MN327643.1 were added to the phylogenetic analysis.

### Clustering and biomarker analysis

Patterns of relative abundance of taxa species in response to different experimental factors were analyzed in MicrobiomeAnalyst. To provide an estimate of the most important taxa for classification of data resulting from different experimental factors, biomarker analysis was carried out using the random forest algorithm within MicrobiomeAnalyst. The default setting of the number of trees to grow and number of predictors to try (500 and 7, respectively) was applied with the randomness setting left on. The random forest algorithm is a supervised classification algorithm of trees created by using bootstrap samples with training data and random feature selection in tree induction. It is an ensemble of unpruned classifications or regression trees trained with the bagging method [33].

### Analysis of the impact of supplemental feeding of *Ae. albopictus* with *E. anophelis aegypti* on their survivability and vector competence for ZIKV

The *Elizabethkingia* strain associated with the *Ae. albopictus* in this study was identified as *E. anophelis albopictus* (Fig. 3). *Elizabethkingia anophelis aegypti* cultured from *Ae. aegypti* midguts was used for supplemental feeding of *Ae. albopictus*, which were reared to adult stage as described previously. Septic adult *Ae. albopictus* were fed a supplemental diet of *E. anophelis aegypti* grown to optical density at 600 nm (OD_600_) = 1.0 [(3.16E+08 colony-forming units per milliliter (CFU/ml)] (*N* = 30) and OD_600_ = 0.5 (1.0E+08 CFU/ml) (*N* = 30), after which the survivability was assessed. Sterile PBS was used as a negative control (*N* = 30), and the differences in survivability were calculated using the Log-rank test.

To assess the impact of *E. anophelis aegypti* supplemental feeding on *Ae. albopictus* vector competence for ZIKV, *Ae. albopictus* mosquitoes were reared to the adult stage, and upon emergence, *Ae. albopictus* adults were antibiotic-treated with 20% penicillin, 20% streptomycin, and 75ug/ml gentamicin in a 10% sucrose solution for 8 days (Fig. 4b). The cotton strips that were used to deliver the antibiotic treatment were changed daily. Both groups were maintained on filter-sterilized 10% sucrose solution. The effectiveness of the sterility rearing was confirmed by CFU assays on randomly selected adult female midguts prior to blood-feeding or bacterial challenge (Fig. 4c). *Elizabethkingia anophelis aegypti* was introduced to a total of *n* = 75 antibiotic-treated *Ae. albopictus* through a sugar meal (cotton strips moistened with 1.5% sucrose solution containing *E. anophelis aegypti* to a final concentration of 1.0E+08 CFU/ml) [9]. The control consisted of septic *Ae. albopictus *(*n* = 75) without exposure to *E. anophelis aegypti*. The cotton strips were withdrawn after 24 h and replaced with sterile cotton strips moistened with sterile 10% sucrose solution. Both the experimental (antibiotic-treated *Ae. albopictus* infected with *E. anophelis aegypti*) and control groups (septic *Ae. albopictus*) were deprived of sucrose solution for 18–24 h and offered 2.00E+08 PFU/ml ZIKV blood meal as described previously. The midgut, carcass, saliva, and legs were obtained from each individual of each population at 7 dpi. RNA was isolated from the carcass, saliva, and legs, and screened for ZIKV. The infection, dissemination, and transmission percentage rates were calculated and compared between the two groups using Chi-square tests. The assay was carried out in two independent experiments.

### Assessment of the broad antiviral effect of *E. anophelis *in vitro

The antiviral effect of *E. anophelis aegypti* was assessed in the Vero and C636 cell lines. A total of 3 ml/well of cells (2.0 × 10^5^ cells/ml) was used to seed six-well plates that were incubated at 37 °C (Vero) and 28 °C (C636) and 5% CO_2_ until they attained 90% confluence. Upon attaining 90% confluence, the experimental wells were inoculated with 75 μl of 1.0E+08 CFU/ml of *E. anophelis aegypti* or *Escherichia coli*, while the control wells were inoculated with either *E. anophelis aegypti* or *E. coli* that were heat-inactivated for 6 h at 90 °C. The experiment was performed on two separate days, and each assay was performed in triplicate.

At 24 h post-infection (hpi) with bacteria (*E. anophelis aegypti* or *E. coli*), Vero cell monolayers were infected at a multiplicity of infection (MOI) of 0.1 infectious particles/cell with ZIKV, CHIKV, or DENV. C636 cells were inoculated with ZIKV at 24 hpi with *E. anophelis aegypti* or *E. coli*. The supernatant was harvested at 24 hpi with the virus. The viral load was compared between the experimental and control supernatants. RNA was extracted from the supernatant using the TRIzol procedure (Thermo Fisher Scientific, Waltham, MA, USA) and the virus quantified by qPCR as described previously, using the primers in Table 1. To assess the effect of timing on virus replication, 75 μl of 8.0 log_10_ CFU/ml of *E. anophelis aegypti* or *E. coli* was co-inoculated with 0.1 MOI of ZIKV. Differences in virus quantities were measured using ANOVA in GraphPad Prism.

In order to assess the cell-independent effect of *E. anophelis aegypti* antiviral activity, U4.4 cells (*Ae. albopictus*) were co-infected with *E. anophelis aegypti* and ZIKV.

The *E. anophelis aegypti* bacterial strain was streaked on Luria–Bertani (LB) plates and incubated in a 10% CO_2_ incubator at 28 °C overnight. A single colony was picked using a sterile plastic loop and used to inoculate 500 ml of sterile LB broth. The inoculated broth medium was incubated overnight at 28 °C in an orbital shaker shaking at 225 rpm. The *E. anophelis aegypti* overnight culture was subsequently diluted to OD_600_ = 1.0 (8.5 log_10_ CFU/ml), OD_600_ = 0.5 (8.0 log_10_ CFU/ml), and OD_600_ = 0.2 (7.7 log_10_ CFU/ml).

Individual wells were seeded with 3 ml of 2 × 10^5^ U4.4 cells/ml suspended in minimum essential medium medium without FBS and incubated at 28 °C for 1 h before adding FBS to each well at a final concentration of 20%. The U4.4 cells were then incubated for 5 days in a 10% CO_2_ incubator at 28 °C. After 5 days, the supernatant was carefully removed, and the plates were infected with *E. anophelis aegypti* as described previously at OD_600_ = 1.0 (8.5 log_10_ CFU/ml), OD_600_ = 0.5 (8.0 log_10_ CFU/ml), and OD_600_ = 0.2 (7.7 log_10_ CFU/ml) for three biological replicates. After 1 h of incubation at 28 °C, 3 ml of M&M media containing 20% FBS was added to each well and incubated at 28 °C.

To determine whether the U4.4 cells were successfully infected with *E. anophelis aegypti*, a single well from each replicate of the different dilutions of *E. anophelis aegypti* infection was harvested at 2 dpi.

The cells were spun at 1000 rpm and washed three times with sterile PBS. After the third wash, 100 µl of the washed cells was used to inoculate 3 ml of LB media that was incubated overnight at 28 °C in an orbital shaker at 225 rpm. The experimental wells were infected with 0.1 MOI ZIKV as described previously, as were the ZIKV quantities. Supernatants were harvested from the two treatments at both 2 dpi and 4 dpi. RNA was extracted from the cells using TRIzol (Thermo Fisher Scientific, Waltham, MA, USA). The ZIKV quantities were measured as described previously.

## Results

### Influence of temperature, blood feeding, and ZIKV infection on microbial profile of *Ae. albopictus*

A total of 12 out of 30 *Ae. albopictus* reared in the L temperature regimen disseminated the virus, with a virus titer that ranged from 1 to 2 log_10_ PFU/ml, while 26 out of 30 individuals reared in the M temperature regimen disseminated the virus, with a virus titer that ranged from 1 to 5 log_10_ PFU/ml (Additional file 5). None of the individuals reared in the L temperature regimen transmitted the virus in the saliva, while three individuals reared in the M temperature regime had positive saliva. The viral titer range in saliva was 1–2 log_10_ PFU/ml (Additional file 6).

Illumina MiSeq 16S *rRNA* sequencing resulted in 6,089,629 reads. The number of reads for individual samples ranged from 531,220 to 34,833, with a mean of 152,448. Both the positive spike-in (PhiX) and negative controls (water and non-template control) had a limited number of sequence reads, ranging from 1620 to 2823, and hence were discarded in downstream analysis. In order to control for alpha and beta diversity biases, each sample was rarefied to a total frequency of 34,000 reads. A total of 1878 OTUs were identified in this study. The core phyla identified were Bacteroidetes, Proteobacteria, Actinobacteria, Cyanobacteria, and Firmicutes.

**Fig. 1:**
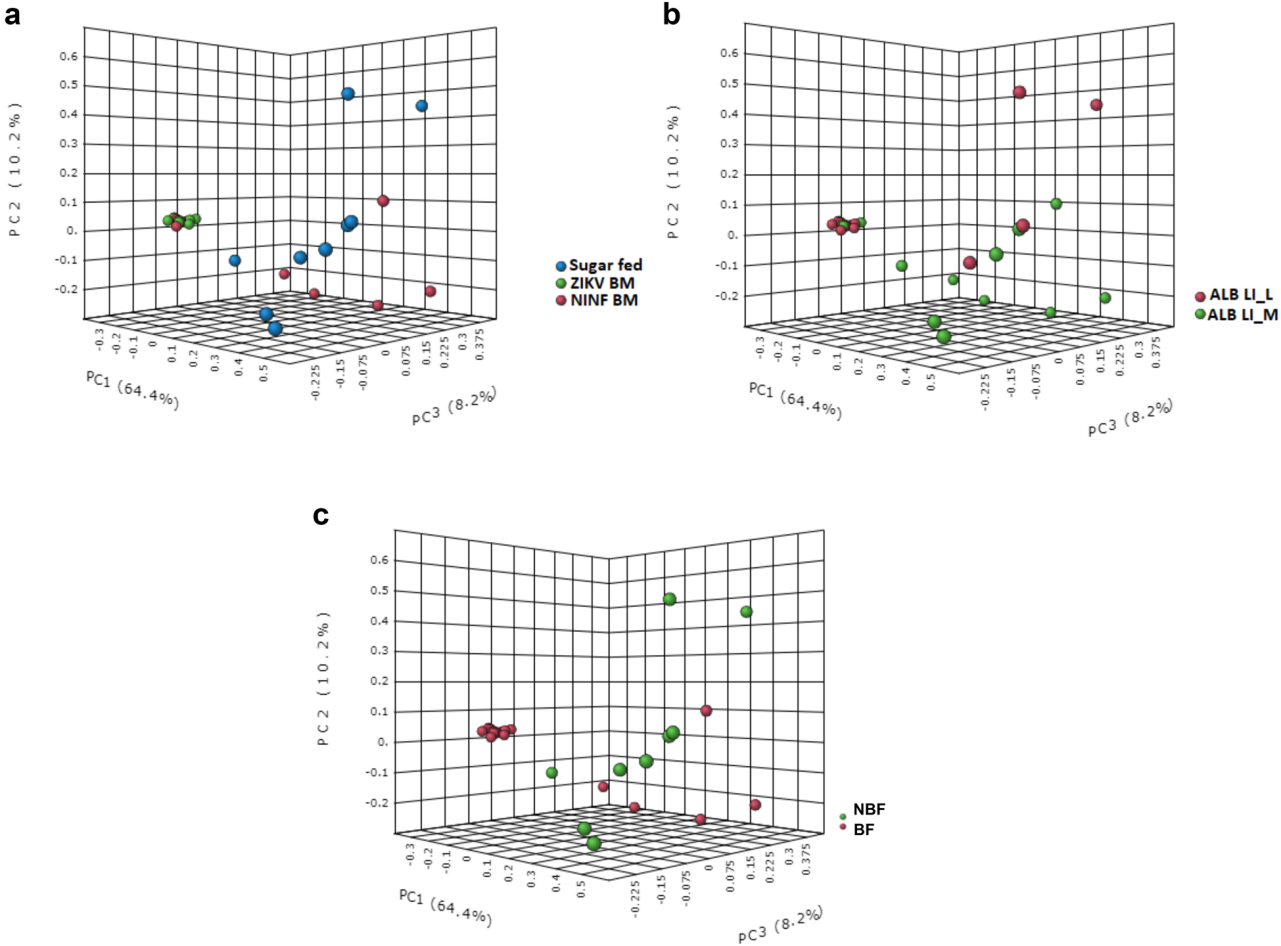
Microbial taxa diversity identified in *Ae. albopictus* midguts. **a** The meal component significantly clustered the midgut taxa [PERMANOVA] *F*-value: 13.22; *R*-squared: 0.5042; *P*-value < 0.001. **b** Increase in temperature did not have an impact on *Ae. albopictus* midgut microbial richness [PERMANOVA] *F*-value: 2.1707; *R*-squared: 0.074415; *P*-value < 0.1. **c** Blood meal intake, irrespective of the infection status, altered the midgut microbial taxa [PERMANOVA] *F*-value: 20.007; *R*-squared: 0.42562; *P*-value < 0.001

**Fig. 2:**
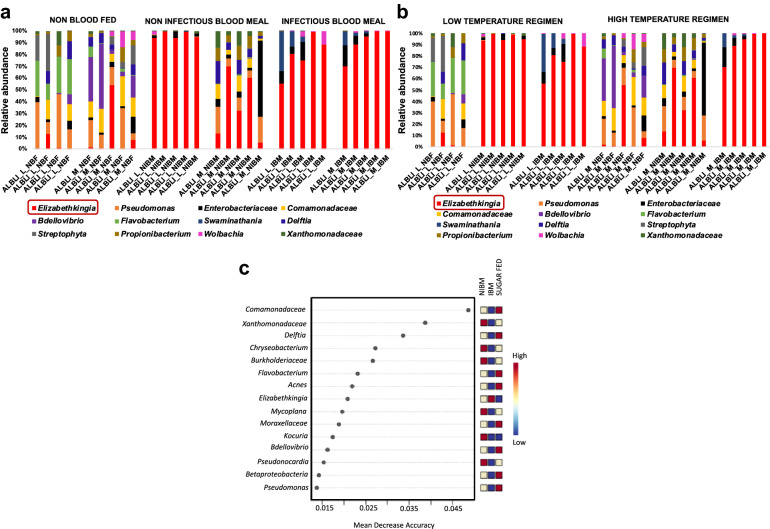
Estimation of relative abundance of bacterial taxa in individual *Ae. albopictus* midguts and association of differently abundant microbial genera with ZIKV infection status. **a** Each stacked bar plot illustrates the taxa distribution at the species level for individual *Ae. albopictus* midguts. The NIBM represent non-infectious blood meal, and IBM are ZIKV-infected BM that acquired an infection, while sugar-fed individuals (non-blood-fed, NBF) were not exposed to blood meal. The L temperature regimen represents day 28 °C and night 24 °C, while M represents day 30 °C and night 26 °C. *Elizabethkingia anophelis albopictus* was abundant among the L temperature regime of NIBM-exposed midguts, as well as in the midguts exposed to IBM. **b** The taxa bar plot is further separated into the L temperature regimen represented by D28N24 (day 28 °C and night 24 °C), and the M temperature regimen represented by D30N26 (day 30 °C/night 26 °C). **c** Mean decrease accuracy is reported for each of the taxa. This measure is obtained by removing the relationship of a taxa and measuring the increase in error. The taxa with highest mean decrease in accuracy is considered to have the highest association with the state. *Elizabethkingia anophelis albopictus* was found to be present in high amounts in the midguts exposed to IBM

Overall, midgut taxa diversity was impacted by feeding [permutational multivariate analysis of variance (Beta-diversity analysis; PERMANOVA test *F*-value = 13.22; *R*-squared = 0.5042; *P*-value < 0.001; Fig. 1a)]. Increasing the rearing temperature did not result in significant changes in taxa diversity (Beta-diversity analysis; PERMANOVA test; *F*-value: 2.1707; *R*-squared: 0.074415; *P*-value < 0.1; Alpha-diversity index: Chao1: *p*-value: 0.85872; *t*-test = 0.17997; *P* = 0.9; Fig. 1b and Additional file 2: Figure S2). Despite this, at the individual taxa level, *Elizabethkingia* was unique in that it was identified at higher proportions at the lower rearing temperature regime (Fig. 2b, Additional file 3: Figure S3). Blood meal intake, irrespective of the infection status, decreased the taxon diversity (PERMANOVA; *F*-value: 20.007; *R*-squared: 0.42562; *P*-value < 0.001; Fig. 1c). Additionally, ZIKV infectious blood meal uptake significantly reduced the individual midgut bacterial diversity (Alpha-diversity index: Chao1; *p*-value: ANOVA test = 24.2116; *p* < 0.05; Fig. 2a; Additional file 4: Figure S4). *Elizabethkingia* was significantly associated with the midgut exposure to an infectious blood meal (Fig. 2c).

### Phylogenetic analysis of *Elizabethkingia*

**Fig. 3:**
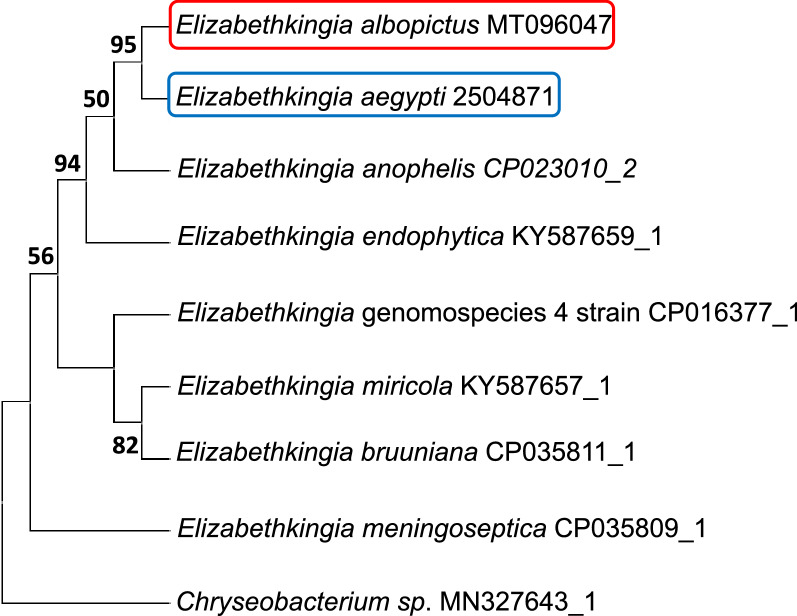
Phylogenetic analysis of the *Elizabethkingia rpoB* gene. Phylogenetic affiliation of partial sequence of the *rpoB Elizabethkingia* taxa gene from the midgut of *Ae. albopictus* (GenBank accession number: MT096047, in red) and *Ae. aegypti* (GenBank submission ID: 2504871 in blue). Bootstrap proportions are shown on branches. The *Elizabethkingia* strain, obtained from Steve Blaire and used in the supplemental feeding of the mosquito as well as in vitro experiments in this study, was 100% homologous to the strain obtained from *Ae. albopictus* as well as *E. anophelis*

To taxonomically classify the *Elizabethkingia* strain that we identified in the midgut of the *Ae. albopictus*, we sequenced the *Elizabethkingia rpoB* gene amplified from the midgut cDNA of *Ae. albopictus*. The phylogenetic analysis demonstrated that the *Elizabethkingia* strain identified in our study has 100% sequence homology to *E. anophelis*, which was originally isolated from the midgut of *Anopheles gambiae* [34]. Our strain has 99% homology to *E. endophytica*, originally isolated from sweet corn [35], and was closely related to the *E. meningoseptica* genomospecies 4 [36], *E. miricola* (isolated from condensation water in the space station) [37], and *E. bruuniana* [38]. *Elizabethkingia anophelis* has 92% homology to *E. meningoseptica*, originally isolated from human cerebrospinal fluid [39] (Fig. 3).

### *Elizabethkingia anophelis* colonization of *Ae. albopictus* midguts is associated with reduced ZIKV infection rates and viral loads

**Fig. 4:**
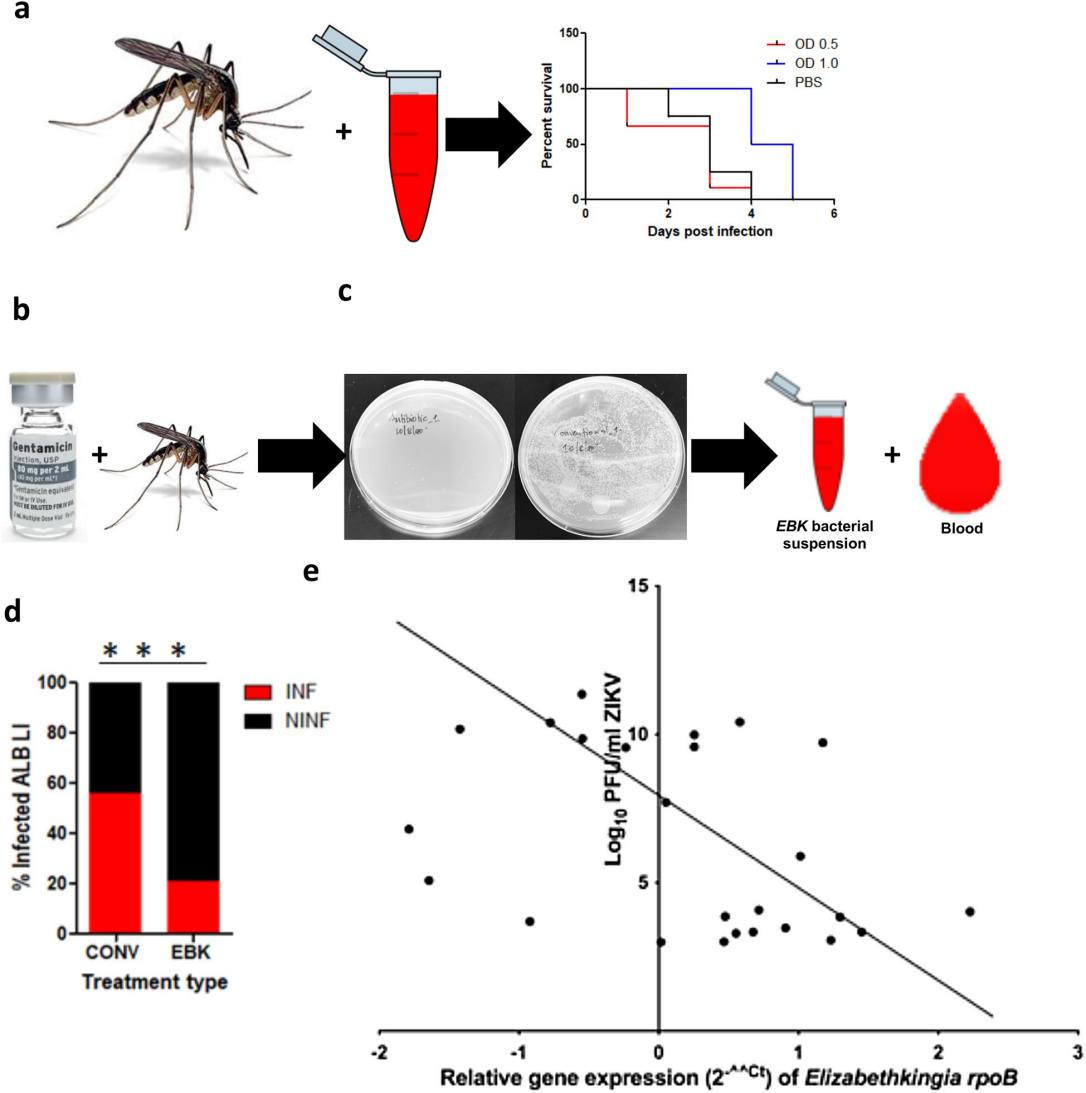
Assessment of the impact of supplemental feeding of *Ae. albopictus* with *EBK* on the infection and dissemination of Zika virus (ZIKV)*.* **a** Impact of *E. anophelis aegypti* artificial infection on the survivability of septic *Ae. albopictus* was assessed at OD_600_ = 1.0 and OD_600_ = 0.5. Survivability across treatments was similar [log-rank (Mantel-Cox) test = 0.4]. **b** Experimental group consisted of *Ae. albopictus* antibiotic-treated with 20% penicillin, streptomycin, and 75ug/ml gentamicin, while the control consisted of conventionally reared *Ae. albopictus*. **c** Bacterial clearance was checked by plating antibiotic-treated midguts on an LB plate. LB plates of antibiotic-treated *Ae. albopictus* midgut did not have any colonies compared to the conventionally reared *Ae. albopictus*. **d** *Aedes albopictus* fed a supplemental diet of *E. anophelis aegypti* and infected with ZIKV demonstrated reduced ZIKV infection rates at 7 dpi (14%), compared to the conventionally reared *Ae. albopictus* (37%) (Fisher’s exact test *P* = 0.0003). **e** A negative correlation between ZIKV and *E. anophelis aegypti* in the midgut was observed (Spearman *r* = −0.3985; *P* < 0.05). The *x*-axis represents log of absolute ZIKV, while the *y*-axis represents log of *E. anophelis aegypti* copy number standards. INF: infected and NINF: non-infected

We assessed the impact of *E. anophelis aegypti* colonization of *Ae. albopictus* midguts on survivability and vector competence for ZIKV. Colonization of *Ae. albopictus* midguts with 3.16E+08 CFU/ml and 1.0E+08 CFU/ml did not impact their survivability [log-rank (Mantel-Cox) test [Chi-square = 2.34*5;* *df* = 2; *P* = 0.31] (Fig. 4a). To study the impact of *E. anophelis aegypti* on the vector competence of *Ae. albopictus* for ZIKV, conventionally reared *Ae. albopictus* was utilized as a positive control; antibiotic-treated *Ae. albopictus* exposed to 1.0E+08 CFU/ml *EBK* constituted the experimental group. The *E. anophelis aegypti*-treated group demonstrated increased blood-feeding rates (40/75) relative to the conventionally reared individuals (19/75) (Chi-square test 12.3207; *P* = 0.0004) (Additional file 7). In spite of the differences in blood meal intake, *E. anophelis aegypti*-treated individuals demonstrated a 14% lower average ZIKV infection rate than the conventionally reared *Ae. albopictus*, which was 37% (Fisher’s exact test, *P* = 0.0003; Fig. 4d). There was no difference observed in the dissemination rates [conventionally reared (5%); *E. anophelis aegypti*-treated (7%) (Fisher’s exact test, *P* = 0.7673)], and no mosquito transmitted the virus at the measured time point (Additional file 8). Despite the fact that feeding on a ZIKV infectious blood meal was associated with a higher percentage of *E. anophelis aegypti* relative to other taxa (Fig. 2a), the fold change in expression of the *Elizabethkingia rpoB* gene from ZIKV-infected *Ae. albopictus* (*N* = 22) correlated negatively with ZIKV absolute quantities (Spearman *r* = −0.3985; *P* < 0.05); (Fig. 4e). ZIKV viral loads ranged from 1.00E+03 to 1.00E+11 ZIKV RNA copies/ml, while the log fold change of *E. anophelis aegypti* ranged from 0.016 to 158.49 CFU/ml (Additional file 9).

### Infection of Vero cells with *E.* anophelis results in a broad-spectrum reduction of viral load

**Fig. 5:**
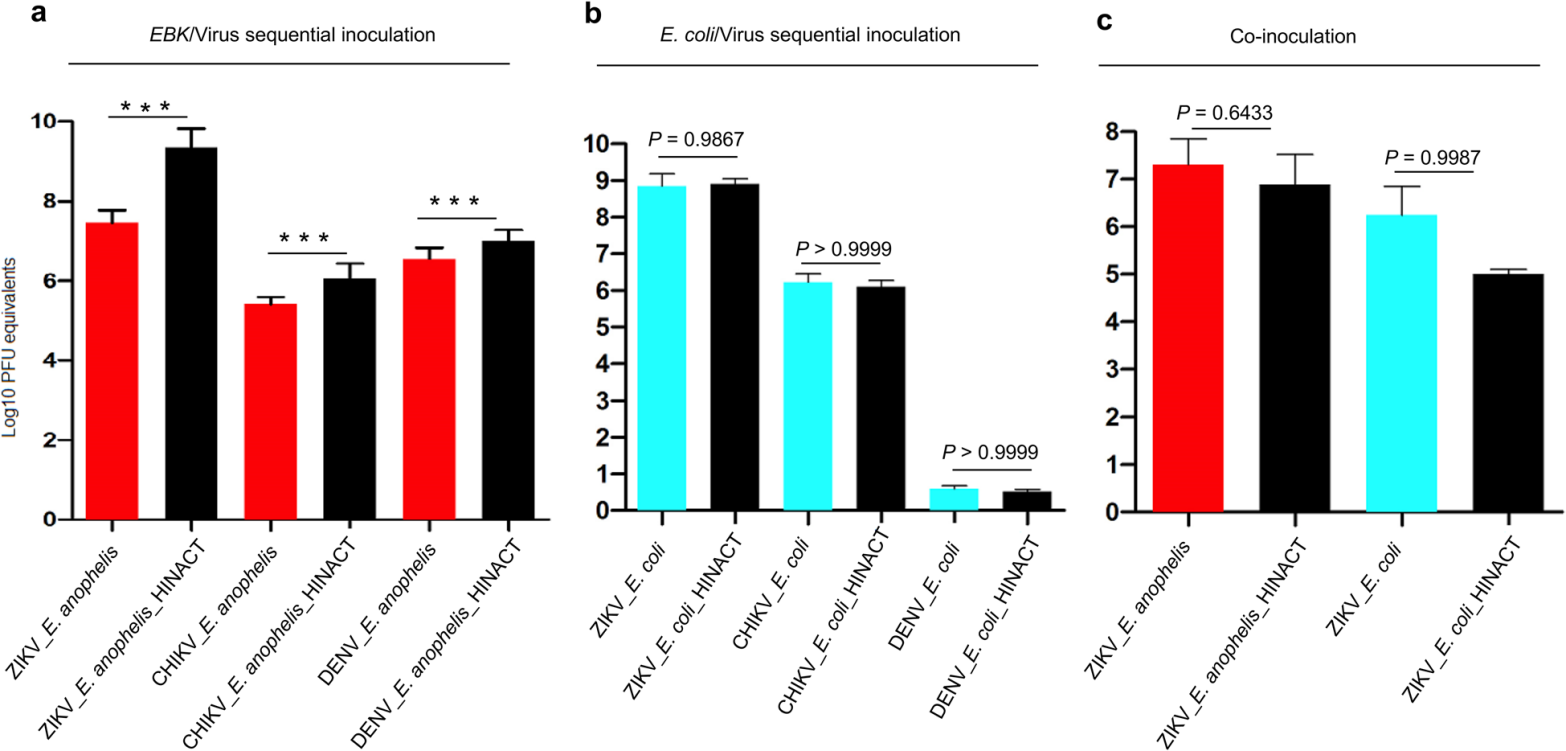
In vitro analysis of the *E. anophelis aegypti* specificity and spectrum of its antiviral effect. **a** Sequential inoculation of Vero cells with *E. anophelis aegypti* for 24 h prior to infection with ZIKV, CHIKV, or DENV resulted in attenuated viral replication of ZIKV (*P* < 0.0001), CHIKV (*P* < 0.0001), and DENV (*P* < 0.0001). **b** Sequential inoculation of Vero cells with *E. coli* and virus did not attenuate viral replication of ZIKV (*P* = 0.9867), CHIKV (*P* > 0.9999), or DENV (*P* > 0.9999), demonstrating the specificity of *E. anophelis aegypti* as an antiviral. **b** Upon co-inoculating Vero cells with ZIKV and *E. anophelis aegypti*/*E. coli*, the antiviral effect was no longer present [ZIKV/*E. anophelis aegypti* (*P* = 0.6433); ZIKV/*E. coli* (*P* = 0.9987)

To clarify the potential for a broad-spectrum antiviral effect of *E. anophelis aegypti*, we performed in vitro assays on both *Ae. albopictus* and mammalian cells. A broad-spectrum *E. anophelis aegypti* antiviral effect was measured by significantly lower viral loads associated with Vero cells sequentially inoculated with *E. anophelis aegypti*/ZIKV (ANOVA test, *P* < 0.0001; Fig. 5a; Additional file 10), *E. anophelis aegypti*/CHIKV (ANOVA test, *P* < 0.0001) (Fig. 5a); (Additional file 10), and *E. anophelis aegypti*/DENV (ANOVA test, *P* < 0.0001; Fig. 5a; Additional file 10).

**Fig. 6:**
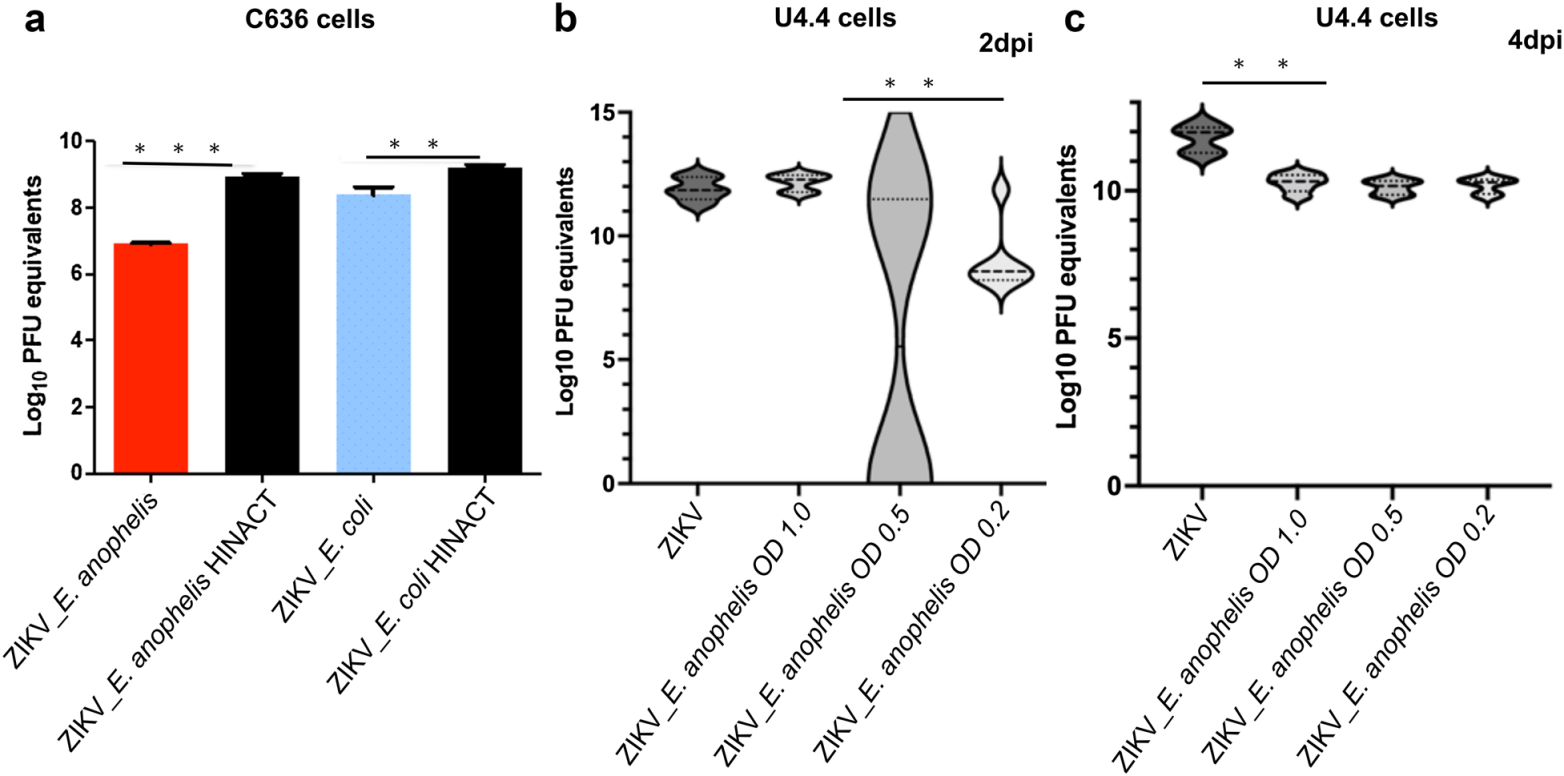
Assessment of the effect of sequential inoculation of C636 and U4.4 cells with *E. anophelis aegypti* and ZIKV. **a** In vitro growth of C636 cells sequentially inoculated with *E. anophelis aegypti* and ZIKV or *E. coli*. ZIKV replication was attenuated in wells co-infected with ZIKV and *E. anophelis aegypti* (*P* < 0.0001) or *E. coli* (*P* < 0.001). **b** In-vitro assay of *E. anophelis aegypti* and Zika virus in U4.4 cells. U4.4 cells were sequentially infected with *E. anophelis aegypti* and ZIKV. We measured a significant reduction in ZIKV titers in all ZIKV/*E. anophelis aegypti*-infected U4.4 cells relative to the control (ANOVA; Kruskal–Wallis test, *P* < 0.01)

This antiviral property was specific to *E. anophelis aegypti*. We demonstrated no difference in viral loads when *E. anophelis aegypti* was replaced by *E. coli*, *E. coli*/ZIKV (ANOVA test, *P* = 0.9867), *E. coli*/CHIKV (ANOVA test, *P* > 0.9999), or *E. coli*/DENV (ANOVA test, *P* > 0.9999; Fig. 5b; Additional file 11). It is noteworthy that the antiviral effect was only measured when *E. anophelis* and the virus were inoculated sequentially, but not when co-inoculated with *E. anophelis aegypti*/ZIKV (ANOVA test, *P* = 0.6433) or *E. anophelis aegypti*/ZIKV (ANOVA test, *P* = 0.9987; Fig. 5c; Additional file 12). When both *E. anophelis aegypti* and *E. coli* were used to inoculate C636 cells, we observed excessive bacteria growth (*E. anophelis aegypti* and *E. coli*) after 24 hpi, and ZIKV replication was attenuated in C636 cells co-inoculated with *E. anophelis aegypti*/ZIKV (ANOVA test, *P* < 0.0001) as well as *E. coli*/ZIKV (ANOVA test, *P* < 0.001; Fig. 6a; Additional file 13). Viability assays demonstrated 90% viability of *E. anophelis aegypti*-infected cells at 2 dpi, suggesting direct cell killing was not responsible for decreased viral replication (Additional file 14). We performed an in vitro assay of *E. anophelis aegypti*/ZIKV on the *Ae. albopictus*-derived U4.4 cell line and measured a significant reduction in ZIKV titers in all *E. anophelis aegypti*/ZIKV-infected U4.4 cells relative to the control (ANOVA; Kruskal–Wallis test, *P* < 0.01; Fig. 6b; Additional file 15).

## Discussion

Recently, ZIKV has become a focus of intense research due to its unprecedented global spread, [4, 40–44] leading to outbreaks in different parts of the world. An upsurge in insecticide resistance, as well as climate change, has resulted in expansion of the geographical range of vector territories [8], which suggests an urgent need for alternative approaches to control mosquitoes and the pathogens they transmit.

Due to their potential as candidates for development of therapeutics and transmission-blocking agents, investigators have studied the interaction of gut microbial taxa and arboviruses of medical importance [9–17]. *Aedes* midgut bacterial species associated with ZIKV are poorly understood. The aim of this study was to assess the impact of increased temperature on the *Ae. albopictus* midgut microbiome and identify bacterial species that are associated with altered ZIKV infectivity and *Ae. albopictus* competence.

### Reduction in abundance of midgut *E. anophelis* due to temperature increase

Given that the global increase in temperature is projected to accelerate [45, 46], a comprehensive assessment of the potential effects of climatic change on vectorial capacity requires an assessment of the influence of temperature on the mosquito microbiome. Studies have shown that temperature is an important component in shaping the microbial communities of organisms [47–51]. In an effort to understand how climate change might ultimately alter microbial taxa that influence vectorial capacity for ZIKV, we measured the effect of increases in temperature on the midgut microbial community. Specifically, we observed a reduction in levels of *E. anophelis albopictus,* which our data suggest is associated with decreased levels of ZIKV and competence. These data suggest that future increases in temperatures could alter microbial profiles and therefore further exacerbate documented increases in transmission resulting directly resulting from increases in viral replication at higher temperatures.

### Impact of ZIKV infection and blood meal on *Ae. albopictus* gut microbiota diversity

A significant reduction in *Ae. albopictus* midgut microbial diversity resulting from ZIKV infection and/or blood meal digestion was evident, yet the effect on individual genera was highly variable. Our results are consistent with the findings of a previous study assessing the total gut microbiota diversity of *Aedes japonicus* and *Aedes triseriatus* following infection with *La Crosse orthobunyavirus*, which revealed higher midgut bacterial richness among the midguts obtained from newly emerged individuals versus lower richness among midguts exposed to viral infection or a non-infectious blood meal [52]. In addition, our study corroborates the finding of a study aimed at assessing the impact of *West Nile virus* infection on the bacterial diversity of *Culex pipiens*, which revealed higher bacterial diversity at the genus level among the unexposed individuals relative to both exposed infected and the exposed uninfected individuals [53].

Mosquitoes exposed to non-infectious blood meals demonstrated substantial increases in the relative abundance of *E. anophelis albopictus* as compared to unfed individuals, clearly indicating that the gut environment is more amenable to this species’ growth following blood meal digestion. This alteration in microbial composition could be beneficial to *Ae. albopictus.* Specifically, *E. anophelis* has been shown to produce OxyR regulon and antioxidants that, in turn, could protect the mosquitoes from the oxidative stress associated with blood digestion. *Elizabethkingia anophelis* has also been shown to possess hemolytic activity and may therefore help facilitate erythrocyte digestion in the mosquito midgut [54].

### *Elizabethkingia anophelis* reduction of viral loads

Our findings demonstrate that the *E. anophelis aegypti* isolated from *Aedes* midguts is associated with broad-spectrum reduction of viral load of in vitro ZIKV, DENV, and CHIKV, as well as a reduction in ZIKV infection rates, when fed as a supplemental diet to *Ae. albopictus*. We also measured a negative correlation between ZIKV and native *E. anophelis albopictus* in the midguts of *Ae. albopictus*. Further, our findings demonstrate a cell-independent effect such that *E. anophelis* aegypti maintains its antiviral activity with both intact and deficient RNA interference responses. Past studies show that *E. anophelis* attenuates *Plasmodium* parasite development [21]. Taken together, these results suggest that *E. anophelis* has the capacity to directly or indirectly elicit a broad antipathogen phenotype. Further studies delineating the potential antiviral mechanism of *E. anophelis* are needed, as this could lead to the development of novel control measures.

We observed higher infection rates in the conventionally reared *Ae. albopictus* relative to the *E. anophelis aegypti*-infected *Ae. albopictus* group. However, this did not translate to increased virus dissemination, which corroborates the results of other studies [55–59]. While these data suggest that *E. anophelis* may primarily act to block viral infection, both the in vitro data and negative correlation of native *E. anophelis* with viral load suggest that increasing doses could further inhibit viral replication and dissemination. An antiviral effect was only measured when *E. anophelis aegypti* and the virus were inoculated sequentially. While the mechanistic basis is unknown, this suggests that establishment and/or replication of *E. anophelis* is required to effectively perturb viral infection and replication.

## Conclusions

To the best of our knowledge, our study provides the first description of broad-spectrum antiviral association of *E. anophelis*. We demonstrate both a negative correlation of *E. anophelis* and ZIKV in *Ae. albopictus* and an association with reduced viral loads of ZIKV, DENV, or CHIKV in vitro. Further, *E. anophelis* was found to be temperature-sensitive, with decreased levels identified at higher temperatures. Together, these findings have significant implications for our understanding of the interactions of bacterial and viral agents in mosquitoes, not to mention how climate change could influence these interactions. Identifying the mechanistic basis for these associations could improve our understanding of disease epidemiology and ultimately inform the development of novel vector control measures.

## Supplementary Information

**Additional file 1: Figure S1.** A standard curve showing *R*^2^, PCR efficiency and slope used to extrapolate viral load.

**Additional file 2: Figure S2.** A box plot file showing the impact of temperature increase on individual midgut diversity.

**Additional file 3: Figure S3.** A biomarker analysis cluster file bearing patterns of relative abundance of taxa species in response to temperature variations.

**Additional file 4: Figure S4.** A box plot file showing the impact of blood meal on the midgut taxa.

**Additional file 5.** Excel file showing the dissemination of ZIKV at 7 dpi by *Ae. albopictus* reared in the L and M temperature regimens.

**Additional file 6.** Excel file showing the transmission of ZIKV at 7 dpi by *Ae. albopictus* reared in the L and M temperature regimens.

**Additional file 7.** Excel file of the blood feeding rates of *Ae. albopictus* supplementarily fed with *E. anophelis aegypti*.

**Additional file 8.** Excel file listing the ZIKV infection rates of *Ae. albopictus* supplementarily fed with *E. anophelis aegypti.*

**Additional file 9.** Excel file of the *Ae. albopictus* ZIKV/*E. anophelis aegypti* correlation analysis.

**Additional file 10.** Excel file of virus titer of sequential inoculation of Vero cells with ZIKV, CHIKV or DENV and *E. anophelis aegypti.*

**Additional file 11.** Excel file of virus titer of sequential inoculation of Vero cells with ZIKV, CHIKV or DENV and *E. coli.*

**Additional file 12.** Excel file of virus titer of co-inoculation of Vero cells with ZIKV and *E. anophelis aegypti* or *E. coli.*

**Additional file 13: Table S9.** An excel sheet of ZIKV titer of sequential inoculation of C636 cells with ZIKV and *E. anophelis aegypti* or *E. coli.*

**Additional file 14: Table S10.** Excel file of Vero cell viability at 24 hpi with *E. anophelis aegypti.*

**Additional file 15: Table S11.** Excel file of ZIKV titer following *in-vitro* assay of *E. anophelis aegypti* and ZIKV in U4.4 cells.

## Data Availability

Datasets generated during the current study are available in GenBank, as well as included in this manuscript.

## Abbreviations

ZIKV: Zika virus
*EBK*: *Elizabethkingia*
DENV: Dengue virus
CHIKV: Chikungunya virus
NYS: New York state
NYSDOH: New York State Department of Health
MD: Mosquito diluent
PBS: Phosphate-buffered saline
IBM: Infectious blood meal
NIBM: Non-infectious blood meal
NBF: Non-blood-fed
NC: Negative control
NTC: Non-template control
PC: Positive control
OTUs: Operational taxonomic units
MOI: Multiplicity of infection
Hpi: Hours post-infection
CFU: Colony-forming units
MEGA: Molecular Evolutionary Genetic Analysis

## References

[1] Faye O, Freire CCM, Iamarino A, Faye O, de Oliveira JVC, Diallo M, Zanotto PMA, Sall AA (2014). Molecular evolution of Zika Virus during Its emergence in the 20th Century. PLoS Negl Trop Dis.

[2] Cook S, Holmes EC (2006). A multigene analysis of the phylogenetic relationships among the flaviviruses (Family: Flaviviridae) and the evolution of vector transmission. Arch Virol.

[3] Chambers T (1990). Flavivirus genome organization, expression, and replication. Annu Rev Microbiol.

[4] Lanciotti RS, Kosoy OL, Laven JJ, Velez JO, Lambert AJ, Johnson AJ, Stanfield SM, Duffy MR (2008). Genetic and serologic properties of Zika virus associated with an epidemic, Yap State, Micronesia, 2007. Emerg Infect Dis.

[5] Petersen LR, Jamieson DJ, Powers AM, Honein MA (2016). Zika Virus. N Engl J Med.

[6] Gulland A (2016). Zika virus is a global public health emergency, declares WHO. BMJ.

[7] Kazmi SS, Ali W, Bibi N, Nouroz F (2020). A review on Zika virus outbreak, epidemiology, transmission and infection dynamics. J Biol Res.

[8] Wells MB, Andrew J (2019). Anopheles salivary gland architecture shapes Plasmodium sporozoite availability for transmission. Am Soc Microbiol.

[9] Ramirez JL, Short SM, Bahia AC, Saraiva RG, Dong Y, Kang S, Tripathi A, Mlambo G, Dimopoulos G (2014). Chromobacterium Csp_P reduces malaria and dengue infection in vector mosquitoes and has entomopathogenic and in vitro anti-pathogen activities. PLoS Pathog.

[10] Ramirez JL, Souza-Neto J, Cosme RT, Rovira J, Ortiz A, Pascale JM, Dimopoulos G (2012). Reciprocal tripartite interactions between the *Aedes aegypti* midgut microbiota, innate immune system and dengue virus influences vector competence. PLoS Negl Trop Dis.

[11] Anglero-Rodriguez Y, Talyuli A, Blumberg B, Kang S, Demby C, Shields A, Carlson J, Jupatanakul N, Dimopoulos G (2017). An Aedes aegypti -associated fungus increases susceptibility to dengue virus by modulating gut trypsin activity. Elife.

[12] Wu P, Sun P, Nie K, Zhu Y, Shi M, Xiao C, Liu H, Liu Q, Zhao T, Chen X, Zhou H, Wang P, Cheng G (2019). A gut commensal bacterium promotes mosquito permissiveness to arboviruses. Cell Host Microbe.

[13] Lindsey ARI, Bhattacharya T, Newton ILG, Hardy RW (2018). Conflict in the intracellular lives of endosymbionts and viruses: a mechanistic look at Wolbachia-mediated pathogen-blocking. Viruses.

[14] Asad S, Hussain M, Hugo L, Osei-Amo S, Zhang G, Watterson D, Asgari S (2018). Suppression of the pelo protein by Wolbachia and its effect on dengue virus in *Aedes aegypti*. PLoS Negl Trop Dis.

[15] Moretti R, Yen PS, Houé V, Lampazzi E, Desiderio A, Failloux AB, Calvitti M (2018). Combining Wolbachia-induced sterility and virus protection to fight *Aedes**albopictus*-borne viruses. PLoS Negl Trop Dis.

[16] Mohanty I, Rath A, Swain SP, Pradhan N, Hazra RK (2019). Wolbachia population in vectors and non-vectors: a sustainable approach towards dengue control. Curr Microbiol.

[17] McLean BJ, Dainty KR, Flores HA, O’Neill SL (2018). Differential suppression of persistent insect specific viruses in trans-infected wMel and wMelPop-CLA Aedes-derived mosquito lines. Virology.

[18] Chen S, Bagdasarian M, Walker ED (2015). *Elizabethkingia anophelis*: molecular manipulation and interactions with mosquito hosts. Appl Environ Microbiol.

[19] Perrin A, Larsonneur E, Nicholson AC, Edwards DJ, Gundlach KM, Whitney AM, Gulvik CA, Bell ME, Rendueles O, Cury J, Hugon P, Clermont D, Enouf V, Loparev V, Juieng P, Monson T, Warshauer D, Elbadawi LI, Walters MS, Crist MB, Noble-wang J, Borlaug G, Rocha EPC, Criscuolo A, Touchon M, Davis JP, Holt KE, Mcquiston JR, Brisse S (2017). Evolutionary dynamics and genomic features of the *Elizabethkingia anophelis* 2015 to 2016 Winconsin outbreak strain. Nat Commun.

[20] Terenius O, Lindh JM, Eriksson-Gonzales K, Bussière L, Laugen AT, Bergquist H, Titanji K, Faye I (2012). Midgut bacterial dynamics in *Aedes aegypti*. FEMS Microbiol Ecol.

[21] Bahia AC, Dong Y, Blumberg BJ, Mlambo G, Tripathi A, Chandra R, Dimopoulos G (2015). Exploring *Anopheles* gut bacteria for *Plasmodium* blocking activity. Environ Microbiol.

[22] Akhouayri IG, Habtewold T, Christophides GK (2013). Melanotic pathology and vertical transmission of the gut commensal *Elizabethkingia meningoseptica* in the major malaria vector *Anopheles gambiae*. PLoS ONE.

[23] Boissière A, Tchioffo MT, Bachar D, Abate L, Marie A, Nsango SE, Shahbazkia HR, Awono-Ambene PH, Levashina EA, Christen R, Morlais I (2012). Midgut microbiota of the malaria mosquito vector *Anopheles gambiae* and interactions with *Plasmodium falciparum* infection. PLoS Pathog.

[24] Wang Y, Gilbreath TM, Kukutla P, Yan G, Xu J (2011). Dynamic gut microbiome across life history of the malaria mosquito *Anopheles gambiae* in Kenya. PLoS ONE.

[25] Ngwa C, Glockner V, Abdelmohsen U, Scheuermayer M, Fischer R, Hentschel U, Pradel G (2013). 16S *rRNA* gene-based identification of *Elizabethkingia meningoseptica* (Flavobacteriales:Flavobacteriaceae) as a dominant midgut bacterium of the Asian malaria vector *Anopheles stephensi* (Diptera: Culicidae) with antimicrobial activities. J Med Entomol.

[26] Villegas LEM, Campolina TB, Barnabe NR, Orfano AS, Chaves BA, Norris DE, Pimenta PFP, Secundino NFC (2018). Zika virus infection modulates the bacterial diversity associated with *Aedes aegypti* as revealed by metagenomic analysis. PLoS One.

[27] Ciota A, Bialosuknia S, Zink S, Brecher M, Ehrbar D, Morrissette M, Kramer LD (2017). Effects of Zika virus strain and *Aedes* mosquito species on vector competence. Emerg Infect Dis.

[28] IPCC: Third Assessment Report. Cambridge, United Kingdom; 2007.

[29] Dhariwal A, Chong J, Habib S, King IL, Agellon LB, Xia J (2017). MicrobiomeAnalyst: a web-based tool for comprehensive statistical, visual and meta-analysis of microbiome data. Nucleic Acids Res.

[30] Chao A (1984). Nonparametric estimation the number of classes in a population. Ann Math Stat.

[31] Bray RJ, Curtis JT (1957). An ordination of the upland forest communities of Southern Wisconsin. Ecol Monogr.

[32] Tamura K, Peterson D, Peterson N, Stecher G, Nei M, Kumar S (2011). MEGA5: molecular evolutionary genetics analysis using maximum likelihood, evolutionary distance, and maximum parsimony methods. Mol Biol Evol.

[33] Liaw A, Wiener M (2002). Classification and regression by randomForest. R news.

[34] Kämpfer P, Matthews H, Glaeser SP, Martin K, Lodders N, Faye I (2011). *Elizabethkingia anophelis* sp. nov., isolated from the midgut of the mosquito *Anopheles gambiae*. Int J Syst Evol Microbiol.

[35] Kämpfer P, Busse HJ, McInroy JA, Glaeser SP (2015). *Elizabethkingia endophytica* sp. nov. isolated from *Zea mays* and emended description of *Elizabethkingia anophelis* Kämpfer et al. 2011. Int J Syst Evol Microbiol..

[36] Holmes B, Steigerwalt AG, Nicholson AC (2013). DNA-DNA hybridization study of strains of *Chryseobacterium*, *Elizabethkingia* and *Empedobacter* and of other usually indole-producing non-fermenters of CDC groups IIc, IIe, IIh and IIi, mostly from human clinical sources, and proposals of *Chryseobacterium be*. Int J Syst Evol Microbiol.

[37] Li Y, Kawamura Y, Fujiwara N, Naka T, Hongsheng L, Huang X, Kobayashi K, Ezaki T (2003). *Chryseobacterium miricola* sp. nov., A novel species isolated from condensation water of space station Mir. Syst Appl Microbiol.

[38] Nicholson AC, Gulvik CA, Whitney AM, Humrighouse BW, Graziano J, Emery B, Bell M, Loparev V, Juieng P, Gartin J, Bizet C, Clermont D, Criscuolo A, Brisse S, McQuiston JR (2018). Revisiting the taxonomy of the genus *Elizabethkingia* using whole-genome sequencing, optical mapping, and MALDI-TOF, along with proposal of three novel *Elizabethkingia* species: *Elizabethkingia bruuniana* sp. nov. *Elizabethkingia ursingii* sp. nov., and Eliz. *Antonie van Leeuwenhoek*. Int J Gen Mol Microbiol.

[39] King E (1959). Studies on a group of previously unclassified bacteria associated with meningitis in infants. Am J Clin Pathol.

[40] Hayes EB (2009). Zika virus outside Africa. Emerg Infect Dis.

[41] Hennessey M, Fischer M, Staples JE (2016). Zika virus spreads to new areas—region of the Americas, May 2015–January 2016. Morb Mortal Wkly Rep.

[42] Zanluca C, Campos V, De MA, Luiza A, Mosimann P, Igor G, Nunes C, Luz K (2015). First report of autochthonous transmission of Zika virus in Brazil. Mem Inst Oswaldo Cruz.

[43] Musso D, Nilles E, Cao-Lormeau V (2014). Rapid spread of emerging Zika virus in the Pacific area. Clin Microbiol Infect.

[44] Duffy MR, Chen T-H, Hancock WT, Powers AM, Kool JL, Lanciotti RS, Pretrick M, Marfel M, Holzbauer S, Dubray C, Guillaumot L, Griggs A, Bel M, Lambert AJ, Laven J, Kosoy O, Panella A, Biggerstaff BJ, Fischer M, Hayes EB (2009). Zika Virus outbreak on Yap Island, Federated States of Micronesia. N Engl J Med..

[45] Vose R, Easterling K, Kunkel A, LeGrande, Wehner M. Temperature changes in the United States. In Clim Sci Spec Rep Fourth Natl Clim Assesment. Edited by Wuebbles D, Fahey D, Hibbard K, Dokken D, Stewart B, Maycock TUS. Global Change Research Program, Washington; 2017:185–206.

[46] Romero-Lankao P, Smith J, Davidson D, Diffenbaugh N, Kinney P, Kirshen P, Kovacs P, Ruiz L, Barros V, Field D, Dokken M, Mastrandrea K, Mach T, Bilir M, Chatterjee M, Ebi K, Estrada Y, Genova R, Girma E, Kissel A, Levy S, MacCracken S, Mastrandrea P, White L (2014). Climate change 2014: impacts, adaptation and vulnerability. Reg Asp Contrib Work Gr II to Fifth Assesment Rep Intergov Panel Clim Chang.

[47] Fuhrman JA, Steele JA, Hewson I, Schwalbach MS, Brown MV, Green JL, Brown JH (2008). A latitudinal diversity gradient in planktonic marine bacteria. Proc Natl Acad Sci.

[48] Lokmer A, Mathias Wegner K (2015). Hemolymph microbiome of Pacific oysters in response to temperature, temperature stress and infection. ISME J.

[49] Ross PA, Wiwatanaratanabutr I, Axford JK, White VL, Endersby-harshman NM, Hoffmann AA (2017). Wolbachia infections in *Aedes aegypti* differ markedly in their response to cyclical heat stress. PLoS Pathog.

[50] Shan H, Deng W, Luan J, Zhang M-J, Zhen Z, Liu S-S, Liu Y-Q (2017). Thermal sensitivity of bacteriocytes constrains the persistence of intracellular bacteria in whitefly symbiosis under heat stress. Environ Microbiol.

[51] Hussain M, Akutse KS, Ravindran K, Lin Y, Bamisile BS, Qasim M, Dash CK, Wang L (2017). Effects of different temperature regimes on survival of *Diaphorina citri* and its endosymbiotic bacterial communities. Environ Microbiol.

[52] Muturi EJ, Bara JJ, Rooney AP, Hansen AK (2016). Midgut fungal and bacterial microbiota of *Aedes triseriatus* and *Aedes japonicus* shift in response to La Crosse virus infection. Mol Ecol.

[53] Zink SD, van Slyke GA, Palumbo MJ, Kramer LD, Ciota AT (2015). Exposure to west nile virus increases bacterial diversity and immune gene expression in culex pipiens. Viruses.

[54] Kukutla P, Lindberg BG, Pei D, Rayl M, Wanqin Y, Steritz M, Faye I, Xu J (2014). Insights from the genome annotation of *Elizabethkingia anophelis* from the malaria vector *Anopheles gambiae*. PLoS ONE.

[55] Diagne CT, Diallo D, Faye O, Ba Y, Faye O, Gaye A, Dia I, Faye O, Weaver SC, Sall AA, Diallo M (2015). Potential of selected Senegalese Aedes spp mosquitoes (Diptera: Culicidae) to transmit Zika virus. BMC Infect Dis..

[56] Chouin-Carneiro T, Vega-Rua A, Vazeille M, Yebakima A, Girod R, Goindin D, Myrielle DR, Lourenco de Oliveira R, Failloux A (2016). Differential susceptibilities of *Aedes aegypti* and *Aedes albopictus* from the Americas to Zika Virus. PLoS Negl Trop Dis..

[57] Diagne CT, Faye O, Guerbois M, Knight R, Diallo D, Faye O, Ba Y, Dia I, Faye O, Weaver SC, Sall AA, Diallo M (2014). Vector competence of *Aedes aegypti* and *Aedes vittatus* (Diptera:Culicidae) from Senegal and Cape Verde Archipelago for West African lineages of Chikungunya Virus. Am J Trop Med Hyg..

[58] Calvez E, Guillaumot L, Girault D, Richard V, O’Connor O, Paoaafaite T, Teurlai M, Pocquet N, Cao-Lormeau V-M, Dupont-Rouzeyrol M (2017). Dengue-1 virus and vector competence of *Aedes aegypti* (Diptera:Culicidae) populations from New Caledonia. Parasit Vectors.

[59] Gaye A, Wang E, Vasilakis N, Guzman H, Diallo D, Talla C, Ba Y, Dia I, Weaver SC, Diallo M (2019). Potential for sylvatic and urban Aedes mosquitoes from Senegal to transmit the new emerging dengue serotypes 1, 3 and 4 in West Africa. PLoS Negl Trop Dis..

[60] Naze F, Le Roux K, Schuffenecker I, Zeller H, Staikowsky F, Grivard P, Michault A, Laurent P, Cao-Lormeau V-M, Dupont-Rouzeyrol M (2009). Simultaneous detection and quantitation of Chikungunya, Dengue and West Nile viruses by multiplex RT-PCR assays and Dengue virus typing using High Resolution Melting. J Virol Methods..

[61] Laurent P, Roux Le K, Grivard P, Bertil G, Naze F, Picard M, Staikowsky F, Barau G, Schuffenecker I, Michault A (2007). Development of a sensitive real-time reverse transcriptase PCR assay with an internal control to detect and quantify chikungunya virus. Clin Chem..

[62] Callahan JD, Wu SL, Dion-schultz A, Mangold BE, Peruski LF, Watts DM, Porter KR, Murphy GR, Suharyono W, King C, Hayes CG (2001). Development and evaluation of serotype- and group-specific fluorogenic reverse transcriptase PCR (TaqMan) assays for dengue virus. J Clin Microbiol..

