# Supplementary figures and images for: Zika virus and temperature modulate Elizabethkingia anophelis in Aedes albopictus
Source: Parasit Vectors. 2021 Nov 12;14:573. doi: 10.1186/s13071-021-05069-7 (PMC8588690; doi:10.1186/s13071-021-05069-7)

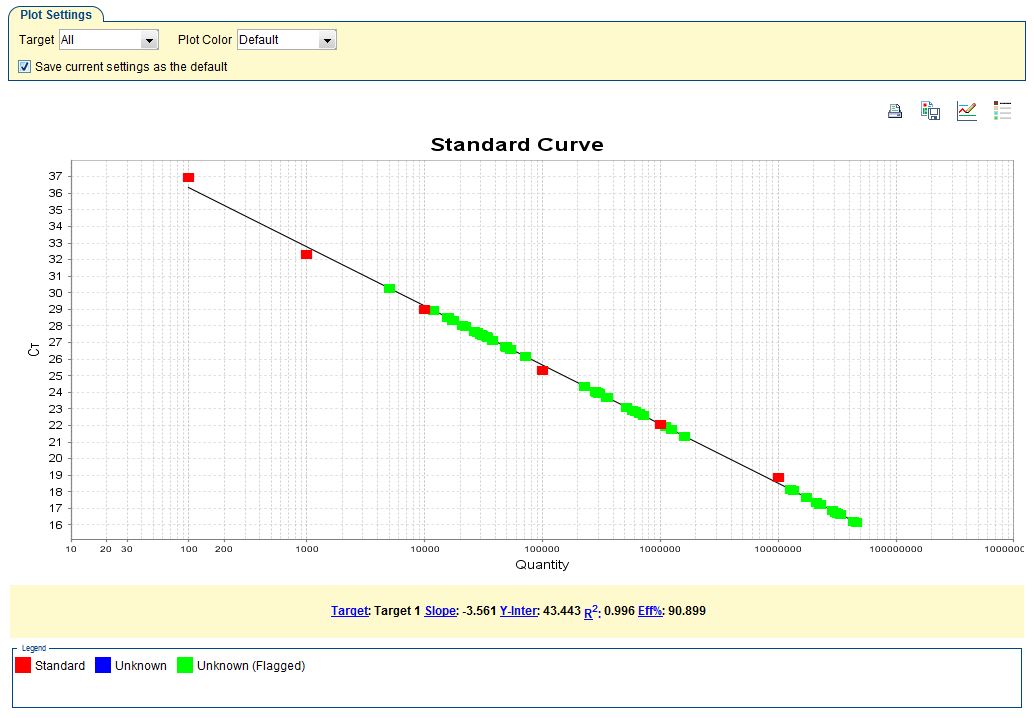

Supplement: Supplementary file 1 — Additional file 1: Figure S1. A standard curve showing R2, PCR efficiency and slope used to extrapolate viral load. [file 13071_2021_5069_MOESM1_ESM.jpg]

Alpha-diversity Index: Chao1

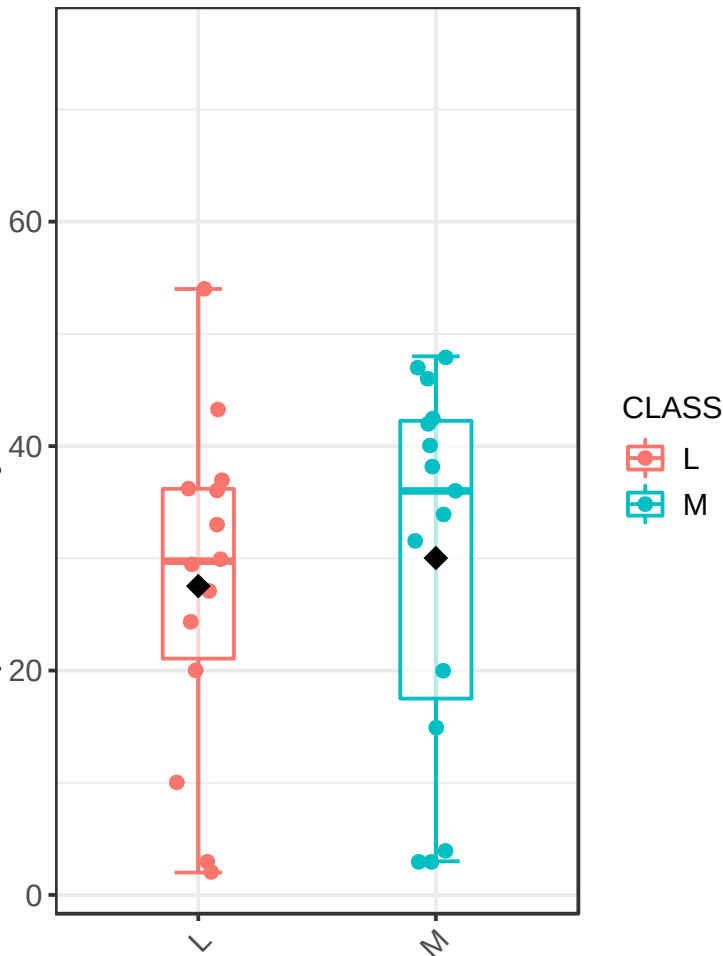

Supplement: Supplementary file 2 — Additional file 2: Figure S2. A box plot file showing the impact of temperature increase on individual midgut diversity. [file 13071_2021_5069_MOESM2_ESM.pdf]

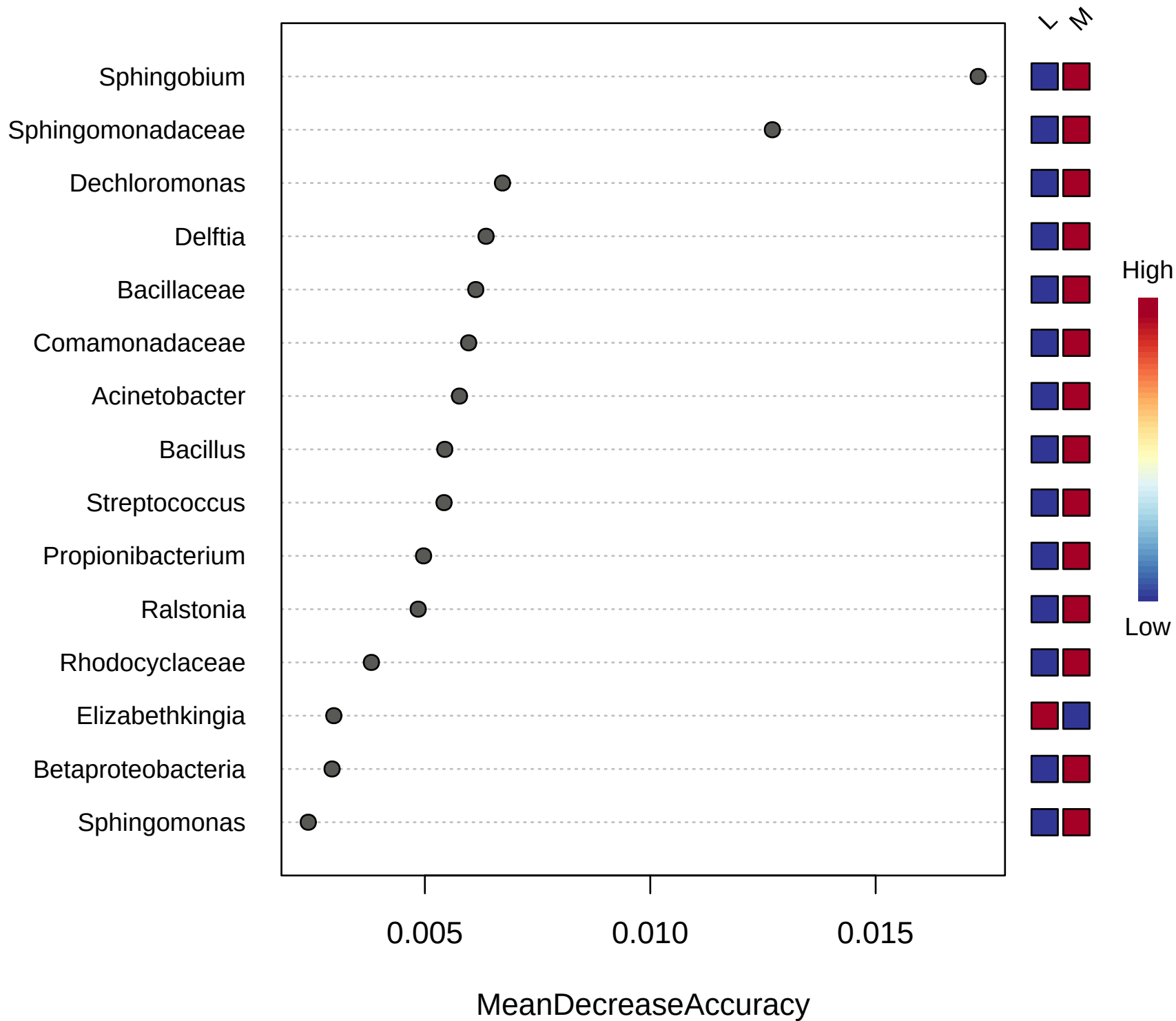

Supplement: Supplementary file 3 — Additional file 3: Figure S3. A biomarker analysis cluster file bearing patterns of relative abundance of taxa species in response to temperature variations. [file 13071_2021_5069_MOESM3_ESM.pdf]

Alpha-diversity Index: Chao1

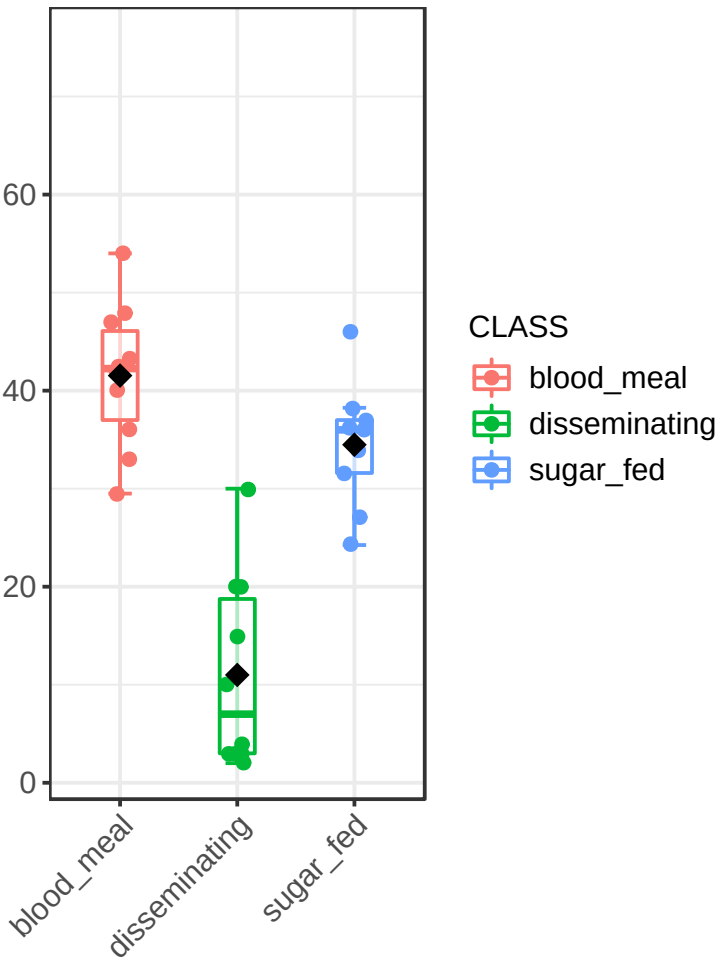

Supplement: Supplementary file 4 — Additional file 4: Figure S4. A box plot file showing the impact of blood meal on the midgut taxa. [file 13071_2021_5069_MOESM4_ESM.pdf]
